# Personality proﬁles in paradoxical insomnia: a case-control study

**DOI:** 10.5935/1984-0063.20190148

**Published:** 2020

**Authors:** Leeba Rezaie, Yasamin Vakili-Amini, Ethan Paschall, Habibolah Khazaie

**Affiliations:** 1 Sleep disorders research center, Kermanshah University of Medical Sciences, Kermanshah, Iran.; 2 Student research committee of Kermanshah University of Medical Sciences, Kermanshah, Iran.; 3 Clinical Psychology Doctoral Fellow, Eastern Michigan University, Ypsilanti, Michigan, USA.

**Keywords:** Paradoxical Insomnia, Psycho-Physiological Insomnia, Sleep Misperception, Personality, Iran

## Abstract

**Introduction:**

Paradoxical insomnia (PARA-I) is a clinically challenging condition to diagnose and treat. Previous ﬁndings suggest that personality proﬁles of patients with PARA-I may be different from other subtypes of insomnia. Therefore, investigation of these proﬁles can be helpful in the clinical management of these patients.

**Objective:**

The current study compares personality proﬁles of individuals with paradoxical insomnia (PARA-I), psycho-physiological insomnia (PSY-I), and normal sleepers (NS).

**Material and Methods:**

A cross-sectional case-control study was conducted in the Sleep Disorders Research Center of Kermanshah University of Medical Sciences, Kermanshah, Iran between 2015 and 2017. Patients with PARA-I (n=20), PSY-I (n=20), and NS (n=60) were matched for age, gender, education, and history of mental and/or physical illness and completed the Minnesota Multiphasic Personality Inventory (MMPI) short form. One-way analysis of variance (ANOVA) and the Kruskal-Wallis test were used to compare subscale means across groups.

**Results:**

With the exception of the schizophrenia scale (P =.059), signiﬁcant differences were found in all subscales of the insomnia groups compared to the NS group (P=.001). Compared to the NS group, patients with PARA-I showed signiﬁcant differences in the hysteria and hypomania subscales (P<.05) and patients with PSY-I showed signiﬁcant differences in the hysteria, hypochondriasis, and psychopathic subscales (P=.001). No signiﬁcant differences were found between the PARA-I and PSY-I groups on any subscale.

**Conclusion:**

This study demonstrates that signiﬁcant differences in the personality proﬁles on the MMPI exist between PARA-I and PSY-I patients compared to NS. These ﬁndings should inform the diagnosis and future treatment approaches for insomnia.

## INTRODUCTION

Insomnia is considered the most common sleep disorder with an estimated prevalence of 10-25% of adults in the general population meeting criteria for the diagnosis^1^. It is defined as the subjective experience of struggling to initiate or maintain sleep, and/or experiencing early morning awakenings that occur at least three times per week for a minimum of three months^2^. Insomnia is associated with several daytime consequences that lead to a decreased quality of life including sleepiness, depressed mood, irritability, cognitive alterations, decreased interest in social activities and impairment in job performance^3-5^. Several classifications exist for insomnia disorders. According to the International Classification of Sleep Disorders, 2nd edition (ICSD-2), there are 11 subtypes of primary insomnia and 2 subtypes: paradoxical insomnia (PARA-I) and psycho-physiological insomnia (PSY-I), which are the most prevalent among other types. The prevalence of PARA-I is estimated to be 9.2-50% of all insomnia patients^6-8^.

PARA-I is also known as “subjective insomnia”, “sleep state misperception” and “pseudo-insomnia.” In this type of insomnia, evident discrepancies exist between the individual’s reports of both sleep quality and quantity compared to objective measures of sleep parameters such as polysomnography (PSG). While misperception about sleep is present in all individuals with insomnia, individuals with PARA-I tend to be at the furthest end of this continuum of misperception^9^, with a previous case report describing the most severe cases as delusional in nature^10^. Individuals diagnosed with PARA-I typically complain that they have not slept, underestimate their total sleep time (TST), and overestimate sleep onset latency and waking after sleep onset^11^. However, PSG reports show normal sleep patterns present in these individuals and a noted lack of daytime sleepiness, which is typically present in cases of sleep deprivation^11^. Therefore, the diagnosis and treatment of this diagnosis is particularly challenging in clinical populations.

Despite the existence of several studies looking at features of PARA-I, the causal mechanism of sleep misperception in these individuals is still not fully understood. Closer examination of the misperceptions and discrepancies between objective measures and subjective reports from individuals with PARA-I is critical, since the subjective discomfort and anxiety, surrounding sleep may eventually progress toward objectively measured sleep difficulties^12^. Conversely, it is possible that individuals diagnosed with PARA-I have comorbid sleep, psychiatric, or medical disorders that may further influence the treatment and prognosis of this disorder^9^.

Two prominent theories prevail regarding PARA-I. The first theory, which emphasizes the neurophysiological mechanisms of sleep misperception, emphasizes the increased cortical arousal and abnormal neuronal circuitry that occurs in these individuals^13^. The second theoretical explanation of PARA-I links insomnia to specific personality traits^13^. Fernandez-Mendoza et al.^14^describe insomnia as a division of two main phenotypes. The first of these phenotypes of insomnia consists of objectively short sleep duration, no misperception, and is associated with poorer neuropsychological performance. The second phenotype is defined as objectively normal sleep duration with clear misperception surrounding sleep. In this second phenotype, insomnia has been associated with Minnesota Multiphasic Personality Inventory 2 (MMPI-2) profiles characterized by depressed mood, rumination, anxiety, intrusive thoughts, and poor resources for coping with stress^14^. The authors of this study stressed that these findings have the potential to be particularly helpful in the diagnosis and treatment of insomnia.

Individuals diagnosed with the first phenotype are likely to respond to treatment aimed at decreasing physiological hyperarousal and increasing sleep duration using medication. Treatment for individuals diagnosed with the second phenotype should aim to decrease cognitive-emotional hyperarousal and misperceptions present using cognitive restructuring and the development of emotion regulation techniques. Therefore, profiles of personality traits can significantly improve the assessment, diagnosis and subsequent treatment of patients with insomnia. Therefore, this case-control study has been designed to compare personality trait profiles in individuals diagnosed with PARA-I, PSY-I and normal sleepers (NS) that do not meet criteria for insomnia.

## MATERIALS AND METHODS

### Study design and setting

A total of 40 participants with insomnia were recruited from patients referred to the Sleep Disorders Research Center of Kermanshah University of Medical Sciences, Kermanshah, Iran, between 2015-2017 for the current case-control study. This facility is the only center in western Iran that focuses primarily on the diagnosis and treatment of sleep disorders.

### Participants

During 2015-2017, 20 patients with PARA-I, 20 patients with PSY-I and 60 NS patients were recruited and matched across groups for age, gender, education, and previous history of mental and/or physical illness. Participants in the insomnia group (which consisted of both PARA-I and PSY-I) were administered subjective measures to assess for previous mental or physical illness and were interviewed by the attending psychiatrist based on diagnostic procedures from the Diagnostic and Statistical Manual of Mental Disorders, fourth edition (DSM-IV).

Additionally, this group of participants were administered an objective measure to examine overnight PSG.

**Figure 1:**
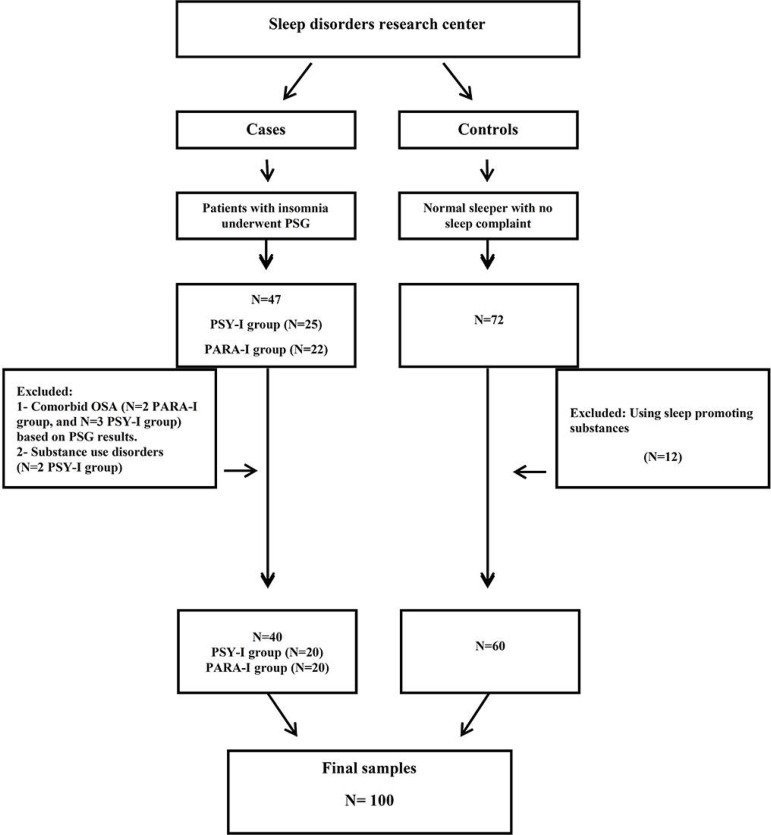
Flowchart of study. Abbreviations: PSG: Polysomnography; PSY-I = Psychophysiological insomnia; PARA-I = Paradoxical insomnia.

In the NS group, only the subjective measures were administered as the high cost of PSG was prohibitive and therefore reserved exclusively to assess the PARA-I and PSY-I groups. In the event of suspected sleep disorders, a different psychiatrist repeated the assessment of NS participants. If the possibility of sleep disorders was detected in an NS participant a second time, the case was removed from the study. Figure 1 shows a flowchart of the study.

Inclusion criteria for all participants were as follows: (a) individuals were required to be over 18-years-old, and (b) individuals were required to have at least a primary school education. Exclusion criteria included: (a) patients with insomnia complaints who were diagnosed with comorbid sleep disorders, such as obstructive sleep apnea (OSA) or periodic limb movement (PLM) based on PSG results; (b) patients with comorbid severe personality disorders; and (c) patients with comorbid substance use disorders. The following sections will describe each study group in detail.

1. **PSY-I group:** Participants in this group were required to meet the following criteria: (a) a subjective complaint of insomnia characterized by difficulties initiating and/or maintaining sleep; (b) insomnia must have been present ≥ 3 nights a week for > 6 months; (c) a complaint of ≥ 1 daytime consequence attributed to insomnia; (d) distress or significant difficulties in social and/or occupational functioning; and (e) sleep efficiency (SE) ≤ 85% as measured by PSG^6,15^.
2. **PARA-I group:** Participants in the PARA-I group were required to meet the same inclusion criteria as those in the PSY-I group, but their objective SE was required to be ≥ 85% and their total sleep time (TST) was required to exceed 390 minutes. Additionally, marked discrepancies between the subjective and objective measures (i.e., a difference of 60 minutes or more of total sleep time and/or a difference of at least 15% between subjective and objective measures of sleep efficiency) were noted and presented as recommended by previous research^6,15^.
3. **NS group:** Participants in the NS group were recruited from the general population by way of advertisements. Participants were required to report sleeping ≥ 7 hours per night, experience satisfaction from their sleep and have no subjective sleep complaints. In addition, to not meeting criteria for insomnia, NS participants were only included if they denied using any sleep-promoting substances.

### Measures and study procedures

1. **PSG:** Overnight PSG techniques were performed for participants in the insomnia group (PARA-I and PSY-I groups) according to the American Academy of Sleep Medicine Manual for the Scoring of Sleep and Associated Events^16^. A SOMNOscreen device was used for PSG measurement called the “SOMNOscreen™ Plus PSG” produced by SOMNOmedics GmbH, Germany. The observed sleep parameters included total sleep time, sleep onset latency, sleep efficiency and wake time after sleep onset.
2. **MMPI:** All participants completed the MMPI short form, which is one of the most frequently used assessment tools for psychopathology^17^and therefore used in the current study to measure personality characteristics. The current study utilized the MMPI short form which has been widely used in Iranian study^17-19^and consists of 71 questions with “yes” or “no” response options. Previous research has determined that the validity and reliability of this measure is satisfactory when used in Iran^20,21^. The MMPI short form is a self-report measure and has three validity scales and eight clinical scales. In the current study, the focus was on the clinical scales: (a) hypochondriasis, (b) depression, (c) hysteria, (d) psychopathic deviate, (e) paranoia, (f) psychasthenia, (g) schizophrenia, and (h) hypomania. Results on the MMPI test are reported as T scores. Therefore, a T score is considered indicative of psychological dysfunction when the value exceeds 70. The current study dichotomously classified each individual participant in terms of whether or not each scale fell in the clinically elevated range (defined as a T score exceeding the cut off of 70). Absolute scores were defined by calculating the mean of T scores.

### Data analysis

The current study used the statistical package SPSS 21.0^22^ for data management and analysis. Data distribution was assessed using the Shapiro-Wilk test and continuous variables are reported as Mean ± SD while categorical variables and are reported as frequencies (%). All sociodemographic variables in the three groups, with the exception of age, were compared using a Chi-square (X^2^) test. One-way analysis of variance (ANOVA) was used to compare the means of participant ages across the three groups. Additionally, ANOVAs and the Kruskal-Wallis test were used to compare the means of personality trait subscales across the three groups. The Tukey and Mann-Whitney U test were also used as post hoc tests. For all results, *P*<.05 was considered statistically significant.

### Ethical approval

The study was approved by the ethical committee of Kermanshah University of Medical Sciences, Kermanshah, Iran, with code number: IR.KUMSREC.1395.22. All procedures performed in studies involving human participants were in accordance with the ethical standards of the committee and based on the 1964 Helsinki declaration and its later amendments or comparable ethical standards. Informed consent was obtained from all participants included in the study.

## RESULTS

In the current study, 100 participants were placed in the following 3 groups: 20 patients with PARA-I, 20 patients with PSY-I and 60 participants who reported normal sleep. In Table 1, the relative frequency and participant demographic variables are reported. Of the 100-participant sample, 56% were women, 60% were university graduates, and 16% had a history of mental and/or physical illness. The results of the Chi-square test showed that the three groups were similar in terms of demographic characteristics and there were no significant differences between them.

**Table 1:** Participant demographic characteristics.

Variable	PSY-I	PARA-I	NS	Total	Statisticsa	*p* value
	(*n = 20*)	(*n = 20*)	(*n = 60*)	(*N = 100*)		
Age (mean±SD)	44.80±11.54	45.30±11.54	44.88±10.73	44.95±10.93	0.794	0.777
Gender n (%)					2.056	
Male	9 (45)	6 (30)	29 (48.33)	44 (44.0)		0.358
Female	11 (55)	14 (70)	31 (51.66)	56 (56.0)		
Education					3. 94	
Diploma	11 (55)	11 (55)	20 (33.3)	30 (50)		0.202
University	9(45)	9 (45)	40 (66.7)	30(50)		
Comorbid illness n (%)					1.559	
Yes	5 (25)	5 (25)	9 (15)	16 (16.0)		0.459
No	15 (75)	15 (75)	51 (85)	84 (84.0)		

Abbreviations: PSY-I = Psychophysiological Insomnia; PARA-I = Paradoxical Insomnia; NS = Normal Sleepers; SD = Standard Deviation, a: Chi-square for all analysis, except age.

The mean ages of PARA-I, PSY-I and NS groups were 44.8±0 range (25-67), 45.3±0 range (27-68), and 44.88±0 range (25-74), respectively. The mean age of all participants in the study was 44.95 with a standard deviation of 10.93 years. The minimum age of participants was 20 years and the maximum age was 74 years. The results of the ANOVA showed no significant differences between the groups in terms of mean age. Therefore, the three groups are considered homogeneous in this regard.

Objective sleep parameters in the two insomnia groups were compared by independent T tests. As see in Table 2, there were significant differences between PARA-I and PSY-I in terms of total sleep time, waking time after sleep onset and sleep efficiency (*P*<.05), but no significant differences in sleep onset latency between these two groups (*P*=.316). The results of the Shapiro-Wilk test showed that the subscales of hysteria, hypochondriasis, psychopathic deviate, and schizophrenia were normally distributed. The remaining subscales (depression, paranoia, hypomania, psychasthenia) were not normally distributed.

**Table 2:** Comparison of objective measure between insomnia groups.

Variable	PSY-I	PARA-I	F	*p* value
	**(mean±SD)**	**(mean±SD)**		
Total sleep time (hours)	4.81±1.83	6.76±.75	19.03	.000
Sleep onset latency (min)	38.58±55.43	22.34±45.02		
			1.64	.316
Wake time after sleep onset (min)	11.88±10.57	3.85±2.81	11.06	.002
Sleep efficiency (%)	63.74±23.21	83.95±20.26	3.14	.004

Abbreviations: PSY-I = Psychophysiological Insomnia; PARA-I = Paradoxical Insomnia; SD = Standard Deviation.

The results are presented in Table 3 and show that 10% of PARA-I and 5% of NS had higher absolute scores in the hysteria scale. Additionally, 35% of PSY-I, 10% of PARA-I, and 5% of NS scored higher on the depression scale. As seen in Table 3, 25% of PSY-I, 10% of PARA-I, and 1.7% of NS had higher absolute scores on the hypochondriasis scale. A higher score on the psychopathic deviate scale was found in 5% of the PSY-I group. Higher scores in paranoia were found in 10% and 25% of the PSY-I and PARA-I groups, respectively, and 1.7% of the NS group. Higher scores on the hypomania scale were seen in 20% of the PSY-I group, 35% of the PARA-I group, and 5% of the NS group. Similarly high scores on the psychasthenia scale were found in the PSY-I and PARA-I groups (35%) while only 5% of scores in the NS group were higher. Both PSY-I and PARA-I groups had higher scores (15%) on the schizophrenia scale, while higher scores were seen in 3.3% of the NS group.

**Table 3:** Comparison of means of MMPI scales among the three groups.

MMPI Clinical Scales	Group	Mean ± SD	*F* (%)	Statistic	*p* value	Effect Size
Hysteria	PSY-I	2.52 ± 2.52	0 (0)	F = 15.41	.001	0.241
	PARA-I	6.90 ± 2.51	2 (10)			
	NS	4.50 ± 2.70	3 (5)			
Depression	PSY-I	10.65 ± 3.89	7 (35)	Kruskal Wallis Test = 22.62	.001	0.228
	PARA-I	10.85 ± 3.37	2 (10)			
	NS	7.10 ± 3.12	3 (5)			
Hypochondriasis	PSY-I	12.75 ± 2.97	5 (25)	F = 14.79	.001	0.234
	PARA-I	12.40 ± 3.16	2 (10)			
	NS	9.31 ± 2.85	1 (1.7)			
Psychopathic Deviate	PSY-I	8.10 ± 2.86	1 (5)	F = 8.4	.001	0.144
	PARA-I	7.58 ± 2.32	0 (0)			
	NS	6.05 ± 2.10	0 (0)			
Paranoia	PSY-I	6.70 ± 2.25	2 (10)	Kruskal Wallis Test = 18.66	.001	0.208
	PARA-I	6.85 ± 2.66	5 (25)			
	NS	4.45 ± 2.11	1 (1.7)			
Hypomania	PSY-I	5.05 ± 2.21	4 (20)	Kruskal Wallis Test = 13.79	.001	0.151
	PARA-I	6.00 ± 2.15	7 (35)			
	NS	3.90 ± 1.96	3 (5)			
Psychasthenia	PSY-I	8.65 ± 4.08	7 (35)	Kruskal Wallis Test = 13.76	.001	0.145
	PARA-I	8.85 ± 3.28	7 (35)			
	NS	5.88 ± 3.31	3 (5)			
Schizophrenia	PSY-I	7.85 ± 4.53	3 (15)	F = 3.65	.059	.070
	PARA-I	8.20 ± 4.26	3 (15)			
	NS	5.90 ± 3.48	2 (3.3)			

Abbreviations: PSY-I = Psychophysiological Insomnia; PARA-I = Paradoxical Insomnia; NS = Normal Sleepers; SD = Standard Deviation.

The results of the ANOVA showed a significant difference between the mean scores of hysteria, hypochondriasis, and psychopathic deviate across the three groups (*P*=.001). Further analysis of the findings and comparison of mean scores with Tukey’s post hoc test identified a significant difference between the mean scores of hysteria, hypochondriasis and psychopathic deviate in the PSY-I group and the NS group (*P*=.001, effect sizes: 0.241, 0.234 and 0.144, respectively). Additionally, there was a significant difference between the mean score of hysteria in the PARA-I and NS groups (*P*=.001). There was no significant difference between the mean score of hysteria personality characteristics in the two PARA-I and PSY-I groups. Additionally, the results of this test yielded no significant differences between the mean scores of the schizophrenia subscale across all three groups.

Analysis using the Kruskal-Wallis test yielded a significant difference between mean scores of depression, paranoia, hypomania and psychasthenia across all three groups (*P*=.001, effect sizes: 0.208, 0.151 and 0.145, respectively). Comparison of the mean differences in groups with the Mann-Whitney U test yielded significant differences in the subscales of depression, paranoia, and psychasthenia between the PARA-I, PSY-I and NS groups (*P*<.05). Significant differences in the hypomania scale were also identified between the PARA-I and NS groups (*P*<.05), but no significant differences were found between the other groups with regard to this subscale.

## DISCUSSION

Significant differences were identified among the PARA-I, PSY-I and NS groups on the subscales of depression, paranoia and psychasthenia (*P*<.05), suggesting that these subscales more accurately assess areas in which these groups differ compared to the hysteria subscale. Increased levels of guardedness, defensiveness and sensation-avoidance have been reported in patients with chronic insomnia, and PSY-I patients specifically exhibit an increased tendency to display these traits^23^.

Surprisingly, the PARA-I group in the current study did not exhibit this tendency, which is inconsistent with these previous studies. This may be partially explained by the tendency of PARA-I patients to overemphasize the severity of their insomnia in spite of the rejection of these claims by their family^4^. The PARA-I and NS groups also demonstrated a significant difference on the hypomania subscale (*P*<.05), which is consistent with previous studies^24^. In summary, it is clear that relevant differences exist when examining the personality profiles of patients with PSY-I, PARA-I and NS.

According to previous studies that examined PARA-I symptom progression, the possibility exists that the subjective discomfort and anxiety surrounding PARA-I could eventually progress toward objectively measured sleep difficulties^12^. Taken together with the results of the current study, these findings suggest that the results of the MMPI short form may differ if administered when symptoms of insomnia first appear. Additionally, this study supports the notions that symptom presentation as captured by the MMPI can be useful in the facilitation of treatment and care to individuals suffering from either PARA-I or PSY-I. Accurate measurement is imperative and closer examination of the significant differences noted in this study has far-reaching implications for future treatment planning by clinicians working with patients suffering from insomnia. The following section will address the ways in which these differences and similarities were highlighted with the goal of informing future studies and treatment of PARA-I and PSY-I patients.

The MMPI short form showed significant differences between mean scores of the insomnia groups and NS groups on the depression, paranoia, hypomania and psychasthenia subscales (*P*=.001, effect sizes: 0.208, 0.151 and 0.145, respectively). These findings are consistent with previous studies that show patients with insomnia tend to have scores in the pathologic range of subscales on the MMPI-2. Examples of pathological traits reported in patients with insomnia include excessive body dissatisfaction, histrionic somatization, and neuroticism^25-27^.

Taking a closer look at the PSY-I group, the results show patients yielding significantly higher reported scores on the hysteria, hypochondriasis and psychopathic deviate subscales when compared to the NS group (*P*=.001, effect sizes: 0.241, 0.234 and 0.144, respectively).

These findings are consistent with reports of other elevated levels of personality traits (pathological hypochondriasis, sensitivity, somatization and internalization) reported in patients with PSY-I^28^. Elevations on the pathological hypochondriasis scale are therefore consistent with previous findings and likely the result of a patient’s experience of insomnia and its daytime consequences. However, previous studies have not reported similar elevations on the hysteria and psychopathic deviate scales. While it is possible that these scales reflect elevations due to the presence of physical and emotional daytime consequences (similar to the elevations of pathological hypochondriasis), it is beyond the scope of this manuscript to identify the underlying cause of this relationship. Therefore, it is recommended that future studies with larger sample sizes and methodological designs that would allow for underlying conclusions to be drawn about the cause of this phenomenon are recommended in order to provide more information regarding these results. In addition to elevations on the hysteria subscale in the PSY-I group, results also showed a significant difference (*P*=.001) between the hysteria scores in the PARA-I and NS groups.

Surprisingly, no significant differences on the hysteria subscale were found when comparing the two PARA-I and PSY-I groups, which is in direct contradiction of van de Laar et al.^29^ proposal that hysteria can be used to accurately differentiate between these two insomnia subtypes. These findings suggest that the hysteria scale on the MMPI short form may be useful in differentiating between patients with insomnia *versus* patients without insomnia, but is less useful in differentiating between the two insomnia subtypes.

No significant differences were found between the three groups on the schizophrenia subscale (*P*=.059). This finding is consistent with previous research that does not report higher scores on the schizophrenia scale^30^. The schizophrenia scale of the MMPI short form is comprised of features like hallucinations, confusion, and limited social interests, which are not typically seen in patients with insomnia.

Although it is impossible to identify causation given the methodology of the current study, it is possible that future studies could seek to answer the question posed by previous studies of whether personality plays a role in the development of insomnia, or if the presence of chronic insomnia affects an individual’s answers on a personality profile questionnaire^31^. Previous research on the direction of this relationship indicates that personality traits in patients with insomnia have the potential to increase the risk of developing future anxiety and depression symptoms^30^. Additionally, a patient’s personality profile could theoretically affect the effectiveness and outcome of treatment for patients with insomnia. In support of this theory, a lower number of pathology identified on the MMPI scales is correlated with better cognitive behavior therapy (CBT) outcomes^32^, while higher scores in hypomania and schizophrenia have been found in patients who failed in treatment^33^. In conclusion, the current findings demonstrate that it is critical for clinicians to assess personality profiles of patients with insomnia. This personality assessment has the potential to not only aid in diagnostic considerations, but also assist in the prediction of an informed prognosis and potential treatment outcome.

## CONCLUSION

Although the results cannot be generalized to all patients with PARA-I or PSY-I, there are two key conclusions and implications warranted. First, the study showed that significant differences across personality subscales of the MMPI short form could be identified across groups of PARA-I, PSY-I and NS individuals. This is particularly useful when considering treatment approaches for insomnia. In all subscales with the exception of schizophrenia, the NS group reported lower levels of the assessed personality traits when compared to the patients with insomnia, suggesting that treatment of insomnia should consider ways in which personality influences insomnia and ways in which insomnia influences personality. Lastly, despite previous reports on specific personality traits in patients with PARA-I, this study did not reveal significant differences between the two insomnia groups. Therefore, clinicians should be aware of variable personality profiles in subtypes of insomnia, especially within the PARA-I subtype. In the future, it may be helpful to develop tailored interventions for these patients. However, further research is warranted to investigate the role of personality profile in the development of PARA-I and PSY-I.

### Strengths and limitations

As the first study to explore the personality profiles of individuals with PARA-I, PSY-I and NS in Iran, in an effort to progress the knowledge and treatment available to individuals living with insomnia in Iran, our study focused on an understudied population. However, there are limitations of this study that should be addressed. First, due to the high cost of PSG, it was not economically feasible to administer objective measures to the NS group in the same way as the two insomnia groups. Secondly, the sample size in the two insomnia groups was smaller than the NS group, which in turn may affect the results. Finally, the study was a cross-sectional study in nature and the role of personality traits in the development and prognosis of PARA-I was not in the scope of this study. Further research is recommended in order to overcome these limitations.
